# Effects of Replacing Fish Meal and Part of Soybean Meal with Enzymatically Hydrolyzed Soybean Meal on Growth Performance, Diarrhea Incidence, and Intestinal Health in Weaned Piglets

**DOI:** 10.3390/vetsci13101021

**Published:** 2026-09-27

**Authors:** Nan Wang, Fu Tang, Yuyilin Nie, Shanyuan Zhang, Yuekai Li, Yong Li, Xiaolong Li, Yu Pi, Xilong Li, Jie Guo, Yanpin Li

**Affiliations:** 1 Key Laboratory of Feed Biotechnology of Ministry of Agriculture and Rural Affairs, Institute of Feed Research, Chinese Academy of Agricultural Sciences, Beijing 100081, China; w13081335110@163.com (N.W.); 13583857992@163.com (Y.N.); piyu@caas.cn (Y.P.); lixilong@caas.cn (X.L.); 2 College of Animal Science and Veterinary Medicine, Shenyang Agricultural University, Shenyang 110866, China; 3 COFCO Feed (Peixian) Co., Ltd., Xuzhou 221600, China; tangfu@cofco.com; 4 COFCO Nutrition and Health Research Institute Co., Ltd., Beijing 102209, China; zhangshanyuan@cofco.com (S.Z.); liyuekai@cofco.com (Y.L.); li-xiaolong@cofco.com (X.L.); 5 COFCO Feed Co., Ltd., Beijing 100020, China; liyong25@cofco.com

**Keywords:** enzymatically hydrolyzed soybean meal, growth performance, diarrhea incidence, intestinal health, weaned piglets

## Abstract

The aim of this study was to evaluate the effects of substituting fish meal and part of soybean meal with enzymatically hydrolyzed soybean meal (EHSM) on growth performance, diarrhea incidence, antioxidant and immune status, and intestinal health in weaned piglets. Eighty 25-day-old piglets were distributed into two treatments with 8 replicate pens of 5 piglets each and fed a control diet or an EHSM diet for 42 days, with EHSM completely replacing fish meal and partially replacing soybean meal at inclusion levels of 9% and 4% during the pre-starter and starter phases, respectively. Compared with controls, EHSM increased day-42 body weight, average daily gain during days 28–42 and overall, and decreased feed conversion ratio during days 0–14 (*p* < 0.05). EHSM also reduced diarrhea incidence during days 0–14 (*p* = 0.050), decreased jejunal crypt depth, elevated jejunal villus height-to-crypt depth ratio, and increased *NQO1* mRNA expression (*p* < 0.05). Plasma antioxidant and immunoglobulin indices were unaffected. Overall, EHSM improved growth and intestinal health, supporting its use as an alternative protein ingredient in weaned piglet diets. Additional research is warranted to clarify how EHSM influences intestinal function in weaned piglets.

## Simple Summary

Fish meal and soybean meal are commonly used protein sources in diets for weaned piglets, but fish meal is expensive and limited in supply, while soybean meal contains antinutritional and antigenic factors. Therefore, finding suitable alternative protein ingredients is important for pig production. In this study, enzymatically hydrolyzed soybean meal (EHSM) was used to completely replace fish meal and partly replace soybean meal in weaned piglet diets. Eighty piglets received either a control diet or an EHSM diet for 42 days. Results showed that replacing fish meal and part of soybean meal with EHSM improved piglet growth, feed utilization, and intestinal health during the post-weaning period. The present observations imply that EHSM is a suitable alternative protein ingredient for weaned piglet diets and may help optimize dietary protein-source combinations while reducing dependence on conventional protein ingredients.

## 1. Introduction

Fish meal is a high-quality animal protein extensively used in weaned piglet diets due to its high protein content, balanced amino acid profile, and superior digestibility, which efficiently supports piglet growth and intestinal health. However, the global decline in fishery resources and the increasing instability of fish meal supply have substantially increased its price and limited its availability, posing major challenges to sustainable swine production [1,2,3,4]. Therefore, identifying sustainable protein ingredients capable of simultaneously reducing dependence on fish meal has become an important objective in modern pig nutrition.

Although various alternative protein sources, including animal protein hydrolysates, fermented plant proteins and insect proteins, have been investigated as substitutes for fish meal, most are restricted by unstable quality, high cost or poor nutritional adaptability for large-scale application [5,6,7,8]. Soybean meal continues to serve as the predominant plant-based protein ingredient in swine production; however, its utilization is restricted by the occurrence of anti-nutritional components, including phytic acid, trypsin inhibitors, and soybean antigen proteins [9,10]. These compounds impair nutrient digestion and uptake, trigger intestinal inflammation and immune responses, and increase susceptibility to post-weaning diarrhea and growth depression. Enzymatically hydrolyzed soybean meal (EHSM), produced through controlled enzymatic hydrolysis, markedly reduces anti-nutritional factors while converting intact proteins into highly digestible small peptides and free amino acids [11,12,13,14]. Owing to its improved nutritional quality and functional properties, EHSM has emerged as a promising protein ingredient for weaned piglets. Earlier studies have shown that appropriate EHSM inclusion can enhance nutrient utilization, improve intestinal health, and alleviate oxidative and immune stress in weaned piglets [15,16].

Previous studies have primarily evaluated EHSM to partially substitute either fish meal or soybean meal in pig diets [17,18,19,20]. However, the effects of EHSM vary depending on the enzymes used and the production process. Moreover, its potential application as a dual protein substitute, together with its overall effects on growth performance and physiological responses in weaned piglets, has not been fully elucidated. Accordingly, this study evaluated dietary replacement of fish meal and part of soybean meal with EHSM, focusing on piglet growth, diarrhea occurrence, and intestinal health. The findings will provide a theoretical basis and technical support for the application of EHSM in optimizing dietary protein structure, reducing feed import dependence, and promoting the efficient and stable production of weaned piglets.

## 2. Materials and Methods

### 2.1. Experimental Design and Diets

A total of 80 healthy 25-day-old weaned piglets (Duroc × Landrace × Large White), with an average initial body weight (BW) of 7.65 ± 0.21 kg, were randomly allocated into two dietary treatments. Each treatment consisted of 8 replicates with 5 pigs per replicate. The feeding experiment lasted 42 days and was divided into two periods: days 0–14 (pre-starter) and days 14–42 (starter). The treatments consisted of a control group fed with a standard corn-soybean meal basal diet and an EHSM diet. In the EHSM diet, fish meal was completely eliminated, and partial soybean meal was synchronously replaced by EHSM. The EHSM inclusion levels were selected based on the nutritional requirements of weaned piglets, the nutritional characteristics of EHSM, and the practical cost of diet formulation. The product was produced from dehulled soybean meal via controlled enzymatic hydrolysis using a multi-enzyme complex (proteases, non-starch polysaccharidases, and phytase) at 38 °C. The process achieved an antigen protein elimination rate exceeding 98% (soybean glycinin: 2.1 mg/g; β-conglycinin: 1.5 mg/g). The product contained 55.2% crude protein, with acid-soluble protein accounting for 17.4% of total protein, and exhibited an in vitro crude protein digestibility of 90.04%. The nutritional composition of the diets was determined according to the NRC (2012) [21]; the specific formulation and nutrient levels of the diets are presented in Table 1.

### 2.2. Animal Management

This study was carried out at the experimental pig farm of the Institute of Feed Science, Chinese Academy of Agricultural Sciences, in Langfang City, Hebei Province, from 17 September–29 October 2025. The pig houses were fully enclosed, with automated control of temperature, humidity, and ventilation rates; ambient temperature was kept at 24–26 °C throughout the trial. Piglets were raised in 1.7 × 1.5 m^2^ pens, each fitted with slatted floors, adjustable stainless steel feeders, and nipple drinkers. Feed and water were provided ad libitum for the duration of the study.

### 2.3. Sample Collection

On experimental day 28, one pig per pen showing the smallest deviation from the mean pen body weight was chosen for both blood and tissue collection to minimize inter-individual variability. Blood was collected from the jugular vein, and the samples were centrifuged at 3000× *g* and 4 °C for 10 min to separate plasma, which was subsequently aliquoted and kept at −20 °C until analysis. Following blood collection, the same pig was electrically stunned and exsanguinated for tissue harvest. Mid-portions of the jejunum and ileum were collected, and 2 cm segments from the center of each middle segment were immersed in 4% paraformaldehyde and subsequently processed for H&E histological examination. The residual intestinal tissues were opened lengthwise and rinsed with sterile physiological saline to remove residual chyme. Following rinsing, the intestinal mucosa was gently removed using sterile glass slides, immediately immersed in liquid nitrogen and subsequently preserved at −80 °C pending analysis.

### 2.4. Growth Performance and Diarrhea Assessment

The BW of individual pigs was recorded biweekly. Daily feed intake per pen was recorded, and residual feed was weighed at each weighing interval. Growth indices, including average daily gain (ADG), average daily feed intake (ADFI), and feed conversion ratio (FCR), were determined for each pen. ADG was calculated at the pen level based on the total body weight gain and total pig-days of all piglets in each pen. When a piglet was culled or died during a given period, its body weight and actual days in the experiment were included in the calculation together with the body weights and pig-days of the remaining piglets. The BW at the end of each period was then calculated for each pen based on the corresponding ADG.

Fecal scores were recorded daily by a trained observer from days 0 to 14 to assess diarrhea incidence. Fecal consistency was evaluated on a 5-point scale: 1, dry and hard pellets; 2, firm and well-formed feces; 3, moist and soft feces retaining their shape; 4, soft feces without a defined shape; and 5, liquid or watery feces. A score of 4 or 5, indicating liquid consistency, was defined as diarrhea. Diarrhea incidence (%) was calculated as: (cumulative diarrheal days in piglets)/(total number of piglets × observation days) × 100.

### 2.5. Plasma Antioxidant Indexes

Plasma activities of catalase (CAT), superoxide dismutase (SOD), and malondialdehyde (MDA) levels were quantified with commercial assay kits purchased from Nanjing Jiancheng Bioengineering Institute (Nanjing, China), with catalog numbers A007-1-1 for CAT, A001-3-2 for SOD, and A003-1-2 for MDA. CAT activity was determined via an ammonium molybdate chromogenic assay, with absorbance measured at 405 nm. SOD activity was determined by a WST-1 non-enzymatic method, which relies on the inhibition of superoxide anion-mediated nitroblue tetrazolium reduction; absorbance readings were taken at 450 nm. MDA concentrations were measured through a thiobarbituric acid (TBA) colorimetric reaction, and absorbance was quantified at 532 nm. All absorbance readings were obtained with an Epoch microplate spectrophotometer manufactured by BioTek Instruments (Winooski, VT, USA).

### 2.6. Plasma Immune Indexes

Porcine-specific ELISA kits (Shanghai Enzyme-Linked Biotechnology Co., Ltd., Shanghai, China) were used to determine plasma concentrations of immunoglobulin A (IgA) and immunoglobulin G (IgG). Serial dilutions of purified recombinant porcine immunoglobulin standards were prepared to establish linear calibration curves for the quantitative calculation of target analyte contents in plasma samples. Each sample was analyzed in duplicate to minimize intra-assay experimental error. The unit of IgA concentration was expressed as μg/mL, whereas IgG contents were reported in mg/mL.

### 2.7. Measurement of Intestinal Morphological Structure

Fixed jejunal and ileal segments were taken out of the fixative before tissue trimming in a fume hood. After trimming, the labeled tissues were transferred to embedding cassettes, subjected to sequential ethanol dehydration and wax infiltration, and subsequently paraffin-embedded. Sections of 4 μm thickness were prepared from the paraffin blocks and subjected to H&E staining before microscopic examination with an OLYMPUS CX43 microscope (Olympus Corporation, Tokyo, Japan). Images were captured using an LV200 digital imaging system (Beijing Changheng Rongchuang Technology Co., Ltd., Beijing, China). Eight non-overlapping fields with intact tissue structure and well-extended villi were then randomly selected and captured. Morphometric measurements were performed with ImageJ v1.54d (National Institutes of Health, Bethesda, MD, USA), from which villus height and crypt depth were obtained and the villus height-to-crypt depth ratio (V:C) was subsequently calculated. All images used for villus morphological measurements were captured with a 10× objective. The measurement scale was calibrated against a standard stage micrometer under the same objective before morphometric analysis.

### 2.8. Real-Time Quantitative PCR Analysis (RT-qPCR)

Frozen jejunal and ileal mucosal samples were subjected to total RNA extraction with TRIzol reagent obtained from Thermo Fisher Scientific, Inc. (Boston, MA, USA), followed by reverse transcription and qPCR analysis as previously described [22]. For cDNA synthesis, 1 μg of total RNA was reverse-transcribed with the PrimeScript RT reagent kit supplied by Takara Biotechnology Co., Ltd. (Dalian, China). qPCR amplification was carried out on a CFX96 Touch system (Bio-Rad Laboratories Inc., Berkeley, CA, USA) with SYBR Green reagent from Thermo Fisher Scientific (Boston, MA, USA). Relative transcript abundance was normalized to *GAPDH* and determined by the 2^−ΔΔCT^ method. Primer information is provided in Table 2.

### 2.9. Statistical Analysis

Growth performance was evaluated in SPSS (version 27.0) with a general linear model (GLM) based on the randomized complete block design. Dietary treatment was included as a fixed effect, with initial body weight serving as the blocking factor, and each pen represented one experimental unit. Diarrhea incidence was evaluated by chi-square analysis, while independent-samples *t*-tests were applied to the other measurements. Values with *p* < 0.05 were regarded as statistically significant, and those with 0.05 ≤ *p* < 0.10 were regarded as indicative of a statistical trend.

## 3. Results

### 3.1. Growth Performance and Diarrhea Incidence

As presented in Table 3, EHSM diet significantly increased the BW of weaned piglets on day 42 compared with the control group (*p* < 0.05). Piglets fed the EHSM diet also exhibited greater ADG during days 28–42 (*p* < 0.05) and across the entire 42-day period (*p* < 0.05). Additionally, EHSM diet decreased FCR during days 0–14 (*p* < 0.05). ADFI, however, remained comparable between treatments throughout the experiment (*p* > 0.05).

EHSM diet lowered the diarrhea incidence of weaned piglets during days 0–14 compared with the control group (88/559 pig-days, 15.74% vs. 112/553 pig-days, 20.25%; Figure 1; *p* = 0.05).

### 3.2. Plasma Antioxidant and Immune Indicators

As indicated in Table 4, EHSM diet had no significant effects on plasma CAT and SOD activities or MDA concentration in weaned piglets (*p* > 0.05).

Similarly, EHSM diet did not significantly affect plasma IgA and IgG levels in weaned piglets (*p* > 0.05).

### 3.3. Intestinal Morphology

Table 5 shows that EHSM diet significantly decreased jejunal crypt depth (*p* < 0.05) and increased the V:C (*p* < 0.05) in weaned piglets. In the ileum, EHSM supplementation tended to increase the V:C (*p* = 0.068), whereas villus height and crypt depth were not significantly affected (*p* > 0.05).

### 3.4. Antioxidant-Related Gene Expression in the Jejunal and Ileal Mucosa

Jejunal *NQO1* mRNA expression was higher in the EHSM group (*p* < 0.05), whereas no significant differences were detected for *Nrf2*, *SOD1*, *CAT*, *GPX1*, and *HO-1* (*p* > 0.05). In the ileal mucosa, none of the antioxidant-related genes examined differed significantly between treatments (Figure 2; *p* > 0.05).

### 3.5. Inflammation-Related Gene Expression in the Jejunal and Ileal Mucosa

EHSM diet had no significant effects on the relative mRNA expression of *TNF-α*, *IL-6*, *IL-8*, *IL-22*, or *IL-1β* in either the jejunal or ileal mucosa of weaned piglets (Figure 3; *p* > 0.05).

### 3.6. Tight Junction-Related Gene Expression in the Jejunal and Ileal Mucosa

Relative mRNA expression of *ZO-1*, *Occludin*, and *Claudin 1* was comparable between the CT and EHSM groups in both jejunal and ileal mucosa (Figure 4; *p* > 0.05).

## 4. Discussion

This study demonstrated that complete replacement of fish meal and partial replacement of soybean meal with EHSM significantly enhances growth performance in weaned piglets. Piglets fed EHSM exhibited improved final BW and ADG throughout the experimental period, along with reduced FCR during the early post-weaning phase. EHSM diet also decreased diarrhea incidence during the first 2 weeks after weaning. These findings indicate that EHSM can support both growth performance and gastrointestinal health when used as a dual substitute for fish meal and soybean meal. The growth-promoting effects observed in the present work agree with earlier reports, which demonstrated that enzymatically modified soybean meal could enhance growth in weaned pigs [5,16,23]. The improved growth performance may be related to the nutritional characteristics of EHSM. Enzymatic hydrolysis reduces several antinutritional and antigenic components of soybean meal and generates readily digestible peptides and amino acids, which may facilitate nutrient utilization during the post-weaning period [9,10,11,14]. This is particularly relevant in newly weaned piglets, whose digestive and absorptive functions are not yet fully developed. However, previous studies have primarily evaluated EHSM as a partial replacement for either fish meal or soybean meal [17,18]. Among the studies reviewed, this study appears to be the first to evaluate EHSM as a dual substitute for the complete replacement of fish meal and partial replacement of soybean meal in diets for weaned piglets. This strategy not only expands the application scope of EHSM in weaned piglet diets but also provides a more comprehensive and cost-effective approach for optimizing dietary protein sources compared to single-substitution strategies reported in prior literature [16,17,18,23]. However, the 42-day experimental period, although sufficient to cover the critical post-weaning transition, does not determine whether the observed improvements in growth performance can be sustained throughout the growing-finishing phase. Future studies with extended feeding periods are therefore warranted to evaluate the sustained effects of this feeding strategy.

In addition to improving growth performance, the EHSM diet exerted beneficial effects on intestinal health in weaned piglets. EHSM reduced jejunal crypt depth and increased the jejunal V:C, indicating improved intestinal morphology and absorptive capacity, which is consistent with previous findings [15,24]. A shallower crypt depth and higher V:C are generally associated with a more favorable intestinal mucosal architecture and potentially improved digestive and absorptive function, which may favor nutrient utilization during the post-weaning period [24]. Thus, the improved jejunal morphology observed in the present study provides a possible physiological link between EHSM supplementation and the improvement in growth performance. These morphological improvements may be partly attributable to the characteristics of enzymatically hydrolyzed soybean protein. Enzymatic hydrolysis reduces anti-nutritional components, including trypsin inhibitors and antigenic proteins, which may alleviate irritation and damage to the intestinal epithelium [9,10,13]. In the present study, the EHSM product used contained only 2.1 mg/g soybean glycinin and 1.5 mg/g β-conglycinin, confirming the effective elimination of antigenic proteins, which may partly account for the improved intestinal morphology observed in EHSM-fed piglets. Enzymatic treatment also breaks down intact proteins into lower-molecular-weight peptides and free amino acids, potentially facilitating their utilization by the immature gastrointestinal tract of weaned piglets [11,14,25]. Consistent with this, the EHSM product used in this study contained approximately 17.4% acid-soluble protein (as a percentage of total crude protein), indicating a substantial proportion of low-molecular-weight peptides that may be rapidly absorbed and contribute to the enhanced intestinal absorptive capacity [11]. Furthermore, jejunal *NQO1* expression was higher in the EHSM group. *NQO1* encodes a phase II detoxifying enzyme involved in cellular antioxidant defense and redox homeostasis and is commonly regulated through the Nrf2 signaling pathway [26,27,28,29]. However, *NQO1* was the only gene showing a significant difference among the 28 gene expression comparisons, and no correction for multiple comparisons was applied. Therefore, this isolated finding should be interpreted cautiously and provides only limited evidence regarding a potential effect of EHSM on intestinal antioxidant responses.

No significant differences were observed in the expression of the other antioxidant-related genes, inflammatory cytokines, or tight junction-related genes in either the jejunal or ileal mucosa. Likewise, plasma antioxidant indices and immunoglobulin concentrations remained unchanged. Taken together, these findings do not support broad alterations in antioxidant, immune, or intestinal barrier-related responses. Additional studies involving more animals are warranted to verify the *NQO1* finding and clarify its biological significance.

From an applied perspective, the dual-substitution strategy evaluated in this study may also help reduce dependence on fish meal and improve flexibility in dietary protein formulation. Collectively, the improvements in growth performance, diarrhea incidence, and intestinal morphology support EHSM as a promising alternative protein ingredient for weaned piglets. However, the relatively small sample size for the physiological and molecular measurements (*n* = 8 per treatment) may have limited the statistical power to detect differences in secondary outcomes, particularly plasma antioxidant and immunoglobulin indices and gene expression. Therefore, these findings should be interpreted with appropriate caution. Further studies with larger sample sizes and extended feeding periods are warranted to confirm these findings and clarify the underlying mechanisms.

## 5. Conclusions

In conclusion, complete replacement of fish meal and partial replacement of soybean meal with EHSM significantly improved growth, enhanced intestinal health, and reduced early post-weaning diarrhea incidence in weaned piglets. These findings indicate that EHSM may serve as a potential alternative protein source under the experimental conditions evaluated.

## Figures and Tables

**Figure 1:**
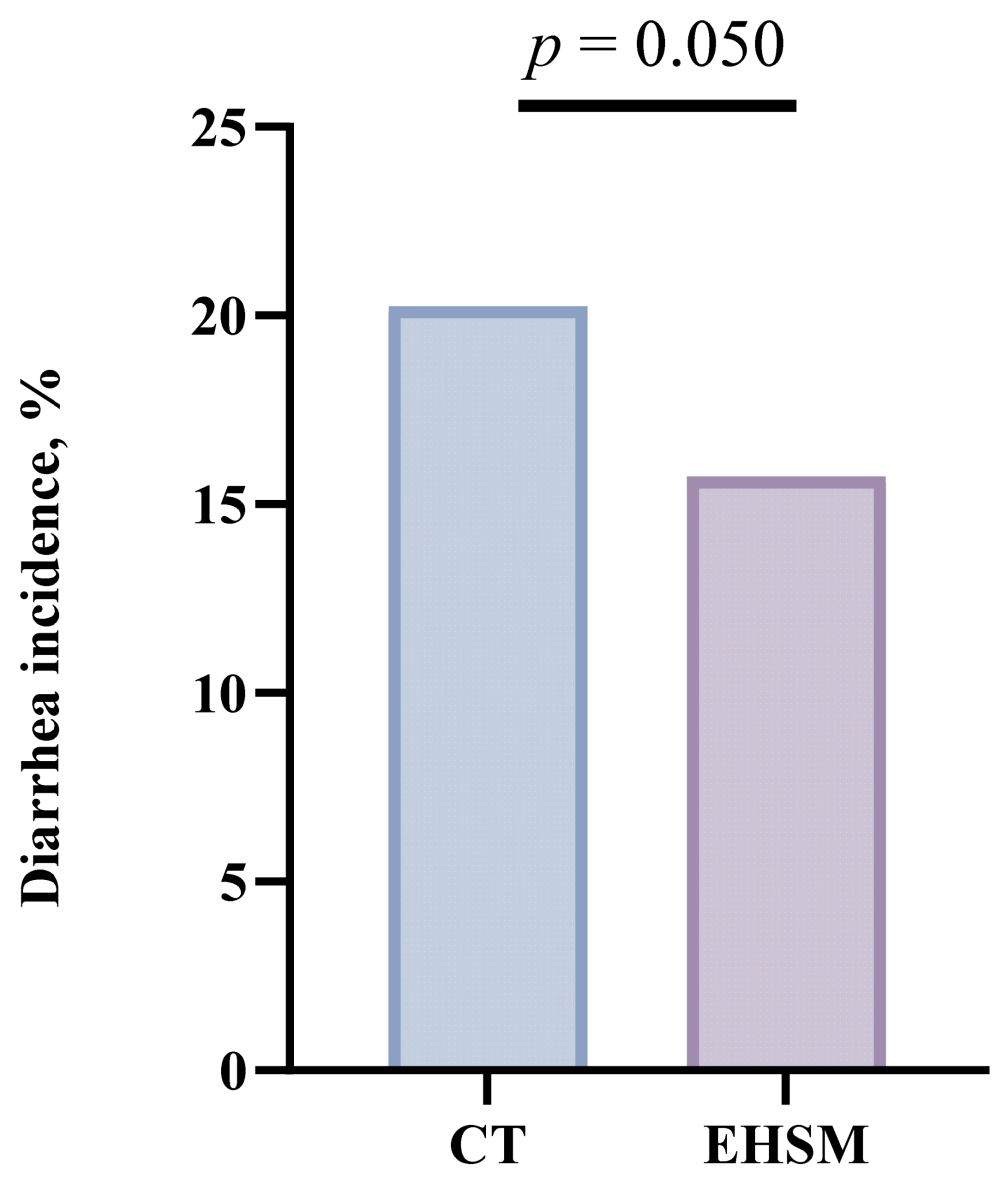
Diarrhea incidence in weaned piglets receiving the CT or EHSM diet during days 0–14. CT = control group, basal diet; EHSM = enzymatically hydrolyzed soybean meal group, fish meal was completely replaced and soybean meal was partially replaced with EHSM at 9% during the pre-starter period and 4% during the starter period.

**Figure 2:**
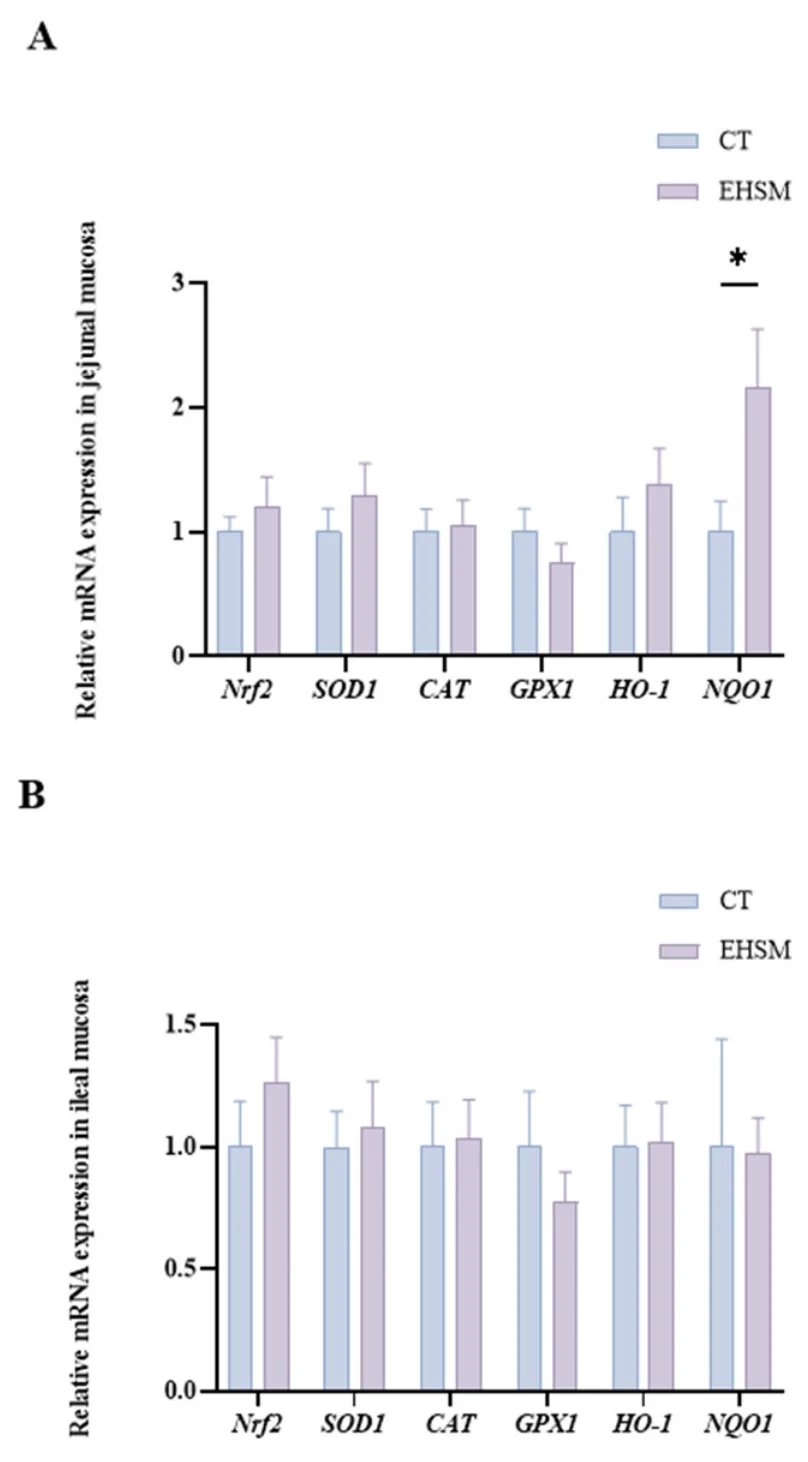
Antioxidant-related gene expression in the jejunal and ileal mucosa of weaned piglets fed the CT or EHSM diet. (**A**) Jejunal mucosa. (**B**) Ileal mucosa. CT = control group, basal diet; EHSM = enzymatically hydrolyzed soybean meal diet, fish meal was completely replaced and soybean meal was partially replaced with EHSM at 9% during the pre-starter period and 4% during the starter period. *Nrf2* = nuclear factor-erythroid 2-related factor 2; *SOD1* = superoxide dismutase 1; *CAT* = catalase; *GPX1* = glutathione peroxidase 1; *HO-1* = heme oxygenase-1; *NQO1* = NAD(P)H: quinone oxidoreductase 1. Data are expressed as mean ± SE, *n* = 8. * *p* < 0.05.

**Figure 3:**
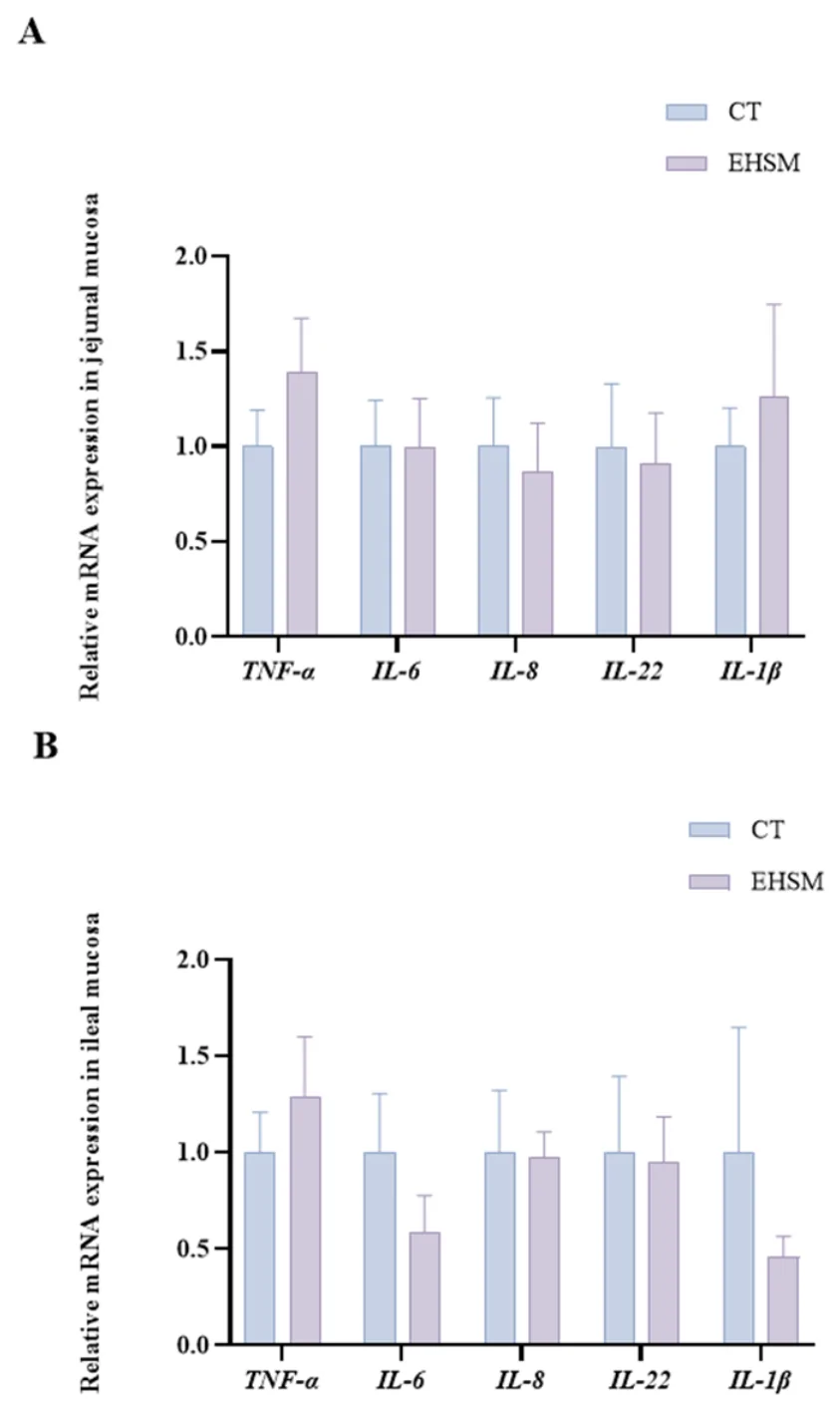
Inflammation-related gene expression in the jejunal and ileal mucosa of weaned piglets fed the CT or EHSM diet. (**A**) Jejunal mucosa. (**B**) Ileal mucosa. CT = control group, basal diet; EHSM = enzymatically hydrolyzed soybean meal diet, fish meal was completely replaced and soybean meal was partially replaced with EHSM at 9% during the pre-starter period and 4% during the starter period. *TNF-α* = tumor necrosis factor-α; *IL-6* = interleukin-6; *IL-8* = interleukin-8; *IL-22* = interleukin-22; *IL-1β* = interleukin-1β. Data are expressed as mean ± SE, *n* = 8.

**Figure 4:**
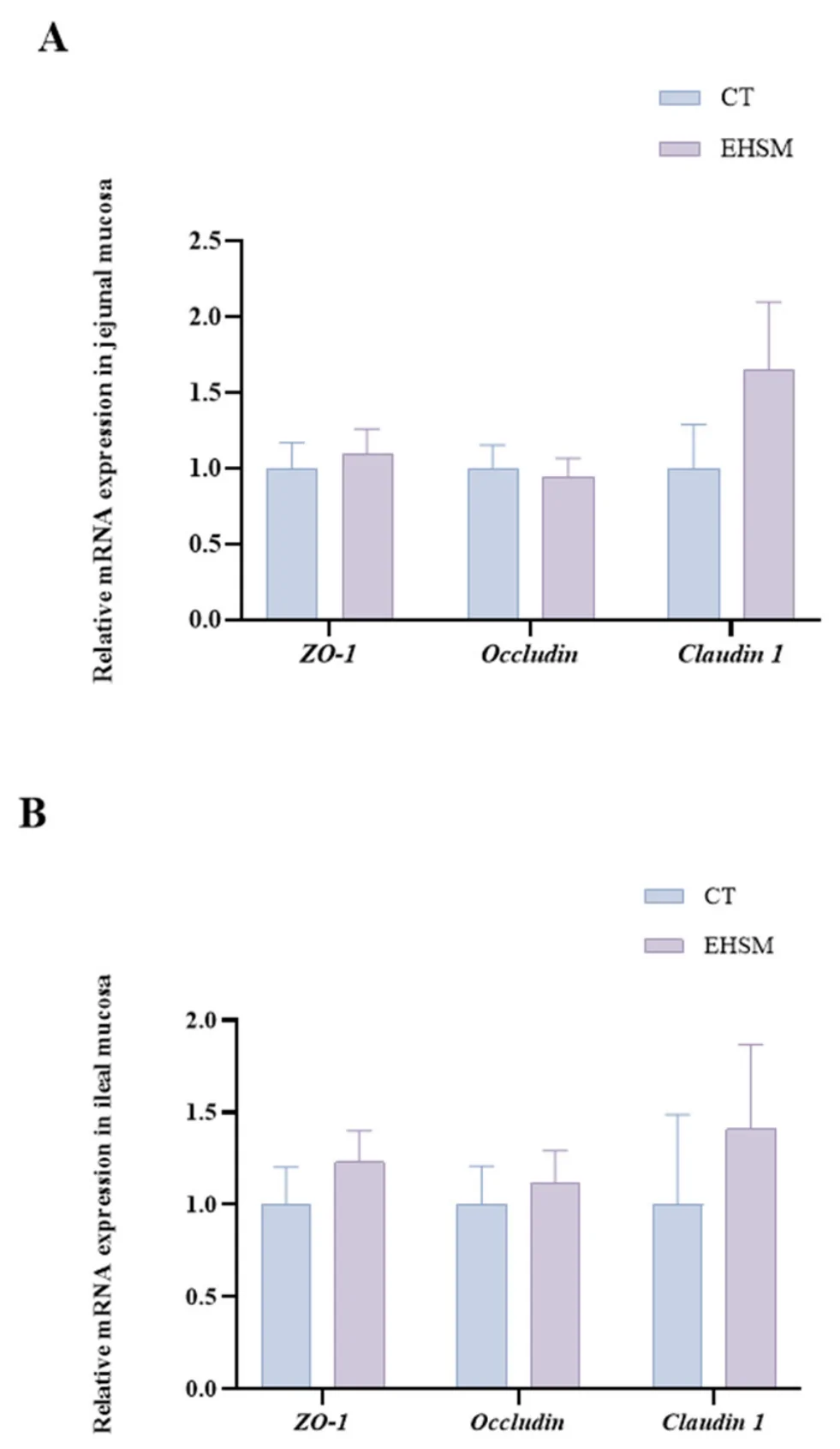
Tight junction-related gene expression in the jejunal and ileal mucosa of weaned piglets fed the CT or EHSM diet. (**A**) Jejunal mucosa. (**B**) Ileal mucosa. CT = control group, basal diet; EHSM = enzymatically hydrolyzed soybean meal diet, fish meal was completely replaced and soybean meal was partially replaced with EHSM at 9% during the pre-starter period and 4% during the starter period. *ZO-1* = zonula occludens-1. Data are expressed as mean ± SE, *n* = 8.

**Table 1:** Ingredients and nutrient composition of diets (as-fed basis).

Items	Pre-Starter (Day 0–14)		Starter (Day 14–42)	
	CT	EHSM	CT	EHSM
Ingredients, %				
Corn	36.89	36.39	52.68	52.92
Extruded corn	15.00	15.00	10.00	10.00
Soybean meal, 46%	16.60	10.20	18.20	14.50
Enzymatically hydrolyzed soybean meal	0.00	9.00	0.00	4.00
Extruded soybean	13.00	13.00	8.50	8.50
Fish meal	3.00	0.00	1.00	0.00
Whey	10.00	10.00	3.00	3.00
Wheat bran	0.00	0.00	1.50	1.50
Soybean oil	0.85	1.20	0.65	0.86
Monocalcium phosphate	0.70	1.00	0.85	0.98
Limestone (CaCO_3_)	0.97	1.18	1.05	1.10
NaCl	0.40	0.40	0.40	0.40
Choline chloride (60%)	0.05	0.05	0.05	0.05
L-Lysine HCl	0.90	0.93	0.75	0.80
DL-Methionine	0.13	0.15	0.11	0.12
Threonine	0.20	0.20	0.16	0.17
Tryptophan	0.04	0.03	0.03	0.03
Phytase	0.02	0.02	0.02	0.02
ZnO	0.20	0.20	0.00	0.00
Feed flavor	0.05	0.05	0.05	0.05
Premix ^1^	1.00	1.00	1.00	1.00
Total	100.00	100.00	100.00	100.00
Analyzed nutrient content				
Crude protein, %	19.37	19.46	18.62	18.59
Calculated nutrient content				
ME, kcal/kg	3400	3400	3350	3350
Calcium	0.80	0.80	0.70	0.70
Phosphorus	0.65	0.65	0.60	0.60
SID Lysine, %	1.30	1.30	1.15	1.15
SID Methionine, %	0.38	0.38	0.34	0.34
SID Threonine, %	0.76	0.76	0.68	0.68
SID Tryptophan, %	0.21	0.21	0.19	0.19

ME = metabolizable energy; CT = control group, basal diet; EHSM = enzymatically hydrolyzed soybean meal group, fish meal was completely replaced and soybean meal was partially replaced with EHSM at 9% during the pre-starter period and 4% during the starter period. ^1^ Premix supplied per kilogram of diet: vitamin A, 11592 IU; vitamin D_3_, 2352 IU; vitamin E, 64 IU; vitamin K_3_, 2.86 mg; vitamin B_1_, 3.42 mg; vitamin B_2_, 6.99 mg; vitamin B_6_, 4.28 mg; vitamin B_12_, 0.038 mg; niacin, 40 mg; calcium pantothenate, 24 mg; folic acid, 6.64 mg; biotin, 0.67 mg; zinc, 79 mg; iron, 150 mg; copper, 23 mg; manganese, 100 mg; iodine, 23 mg; cobalt, 1 mg; selenium, 0.5 mg.

**Table 2:** Primer sequences employed in quantitative real-time PCR.

Gene	Forward (5′-3′)	Reverse (5′-3′)	Product Length, bp	Accession No.
*GAPDH*	GCTTGTCATCAATGGAAAGG	CATACGTAGCACCAGCATCA	86	NM_001206359.1
*Nrf2*	GACCTTGGAGTAAGTCGAGA	GGAGTTGTTCTTGTCTTTCC	103	XM_005671981.3
*SOD1*	GAAGACAGTGTTAGTAACGG	CAGCCTTGTGTATTATCTCC	93	NM_001190422.1
*CAT*	CCTGCAACGTTCTGTAAGGC	GCTTCATCTGGTCACTGGCT	72	NM_214301.2
*GPX1*	TCTCCAGTGTGTCGCAATGA	TCGATGGTCAGAAAGCGACG	104	NM_214201.1
*HO-1*	GAGAAGGCTTTAAGCTGGTG	GTTGTGCTCAATCTCCTCCT	74	NM_001004027.1
*NQO1*	GGACATCACAGGTAAACTGA	TATAAGCCAGAGCAGTCTCG	68	NM_001159613.1
*TNF-α*	TGGCCCCTTGAGCATCA	CGGGCTTATCTGAGGTTTGAGA	68	NM_214022
*IL-6*	AATGTCGAGGCTGTGCAGATT	TGGTGGCTTTGTCTGGATTCT	82	NM_214399.1
*IL-8*	CCGTGTCAACATGACTTCCAA	GCCTCACAGAGAGCTGCAGAA	75	NM_213867.1
*IL-22*	TGGCCCTGAAGAATGAATGC	CTGTGAAGTTTGGCTTCCCAT	139	KX588234.1
*IL-1β*	CAAGGAGATGATAGCAACAA	CATCACACAAGACAGGTACA	87	NM_214055.1
*Occludin*	TCAGGTGCACCCTCCAGATT	TGGACTTTCAAGAGGCCTGG	112	NM_001163647.2
*ZO-1*	CGATCACTCCAGCATACAAT	CACTTGGCAGAAGATTGTGA	111	CV870309
*Claudin 1*	CCTCAATACAGGAGGGAAGC	CTCTCCCCACATTCGAGATGATT	76	NM_001244539.1

*GAPDH* = glyceraldehyde-3-phosphate dehydrogenase; *Nrf2* = nuclear factor-erythroid2-related factor 2; *SOD1* = superoxide dismutase 1; *CAT* = catalase; *GPX1* = glutathione peroxidase 1; *HO-1* = heme oxygenase-1; *NQO1* = NAD(P)H: quinone oxidoreductase 1; *TNF-α* = tumor necrosis factor-α; *IL-6* = interleukin-6; *IL-8* = interleukin-8; *IL-22* = interleukin-22; *IL-1β* = interleukin-1β; *ZO-1* = zonula occludens-1.

**Table 3:** Growth performance of weaned piglets fed the CT or EHSM diet ^1^.

Items	CT	EHSM	*p*-Value
BW, kg			
Day 0	7.65 ± 0.308	7.64 ± 0.300	0.393
Day 14	11.34 ± 0.439	11.54 ± 0.350	0.454
Day 28	14.62 ± 0.546	15.16 ± 0.524	0.141
Day 42	21.21 ± 1.083	23.03 ± 1.275	0.017
ADG, g			
Day 0–14	263 ± 17.3	278 ± 14.1	0.413
Day 14–28	220 ± 20.3	254 ± 23.5	0.249
Day 28–42	445 ± 42.1	500 ± 46.9	0.032
Day 0–42	309 ± 23.4	344 ± 19.7	0.020
ADFI, g			
Day 0–14	362 ± 18.3	343 ± 14.1	0.309
Day 14–28	390 ± 37.9	458 ± 32.8	0.168
Day 28–42	814 ± 61.3	846 ± 59.6	0.657
Day 0–42	522 ± 29.4	549 ± 26.1	0.134
FCR			
Day 0–14	1.39 ± 0.035	1.24 ± 0.039	0.046
Day 14–28	1.80 ± 0.127	1.85 ± 0.106	0.826
Day 28–42	1.88 ± 0.093	1.76 ± 0.139	0.456
Day 0–42	1.71 ± 0.042	1.61 ± 0.053	0.118

BW = body weight; ADG = average daily gain; ADFI = average daily feed intake; FCR = feed conversion rate; CT = control group, basal diet; EHSM = enzymatically hydrolyzed soybean meal group, fish meal was completely replaced and soybean meal was partially replaced with EHSM at 9% during the pre-starter period and 4% during the starter period. ^1^ Eight replicates per treatment (*n* = 8).

**Table 4:** Plasma antioxidant and immunoglobulin indicators of weaned piglets fed the CT or EHSM diet ^1^.

Items	CT	EHSM	*p*-Value
Antioxidant indicators			
CAT, U/mL	4.49 ± 0.262	5.46 ± 0.748	0.252
SOD, U/mL	20.29 ± 0.376	20.22 ± 0.270	0.874
MDA, U/mL	2.07 ± 0.079	2.02 ± 0.124	0.756
Immunoglobulin levels			
IgA, μg/mL	818 ± 23.7	808 ± 21.3	0.776
IgG, mg/mL	22.04 ± 1.603	23.12 ± 1.222	0.601

CAT = catalase; SOD = superoxide dismutase; MDA = malondialdehyde; IgA = immunoglobulin A; IgG = immunoglobulin G; CT = control group, basal diet; EHSM = enzymatically hydrolyzed soybean meal group, fish meal was completely replaced and soybean meal was partially replaced with EHSM at 9% during the pre-starter period and 4% during the starter period. ^1^ Eight replicates per treatment (*n* = 8).

**Table 5:** Intestinal morphology of weaned piglets fed the CT or EHSM diet ^1^.

Items	CT	EHSM	*p*-Value
Jejunum			
Villus height, μm	493 ± 34.52	459 ± 9.00	0.376
Crypt depth, μm	189 ± 11.27	153 ± 4.15	0.010
V:C	2.63 ± 0.156	3.01 ± 0.082	0.047
Ileum			
Villus height, μm	386 ± 9.98	391 ± 20.08	0.830
Crypt depth, μm	165 ± 10.87	141 ± 9.10	0.116
V:C	2.41 ± 0.155	2.81 ± 0.134	0.068

V:C = villus height-to-crypt depth ratio; CT = control group, basal diet; EHSM = enzymatically hydrolyzed soybean meal group, fish meal was completely replaced and soybean meal was partially replaced with EHSM at 9% during the pre-starter period and 4% during the starter period. ^1^ Eight replicates per treatment (*n* = 8).

## Data Availability

The original contributions presented in this study are included in the article. Further inquiries can be directed to the corresponding authors.

## Abbreviations

The following abbreviations are used in this manuscript:

EHSM	Enzymatically hydrolyzed soybean meal
BW	Body weight
ADG	Average daily gain
ADFI	Average daily feed intake
FCR	Feed conversion ratio
ME	Metabolizable energy
H&E	Hematoxylin and eosin
CAT	Catalase
SOD	Superoxide dismutase
MDA	Malondialdehyde
TBA	Thiobarbituric acid
IgA	Immunoglobulin A
IgG	Immunoglobulin G
V:C	Ratio of villus height and crypt depth
GLM	General Linear Model
